# Amputation of the Right Arm at the Shoulder Joint, with Incision of a Portion of the Shoulder Blade for a Necrosis

**Published:** 1870-04

**Authors:** W. H. Hess

**Affiliations:** Nebraska City, Nebraska


ARTICLE IV.—*Amputation of the Right Arm at the Shoulder Joint, with excision of a portion of the Shoulder Blade, for Necrosis.* By W. H. HESS, M.D., Nebraska City, Nebraska.

H. A. GRAHAM, aged twenty-eight years, a soldier in the late war, received a gunshot wound in the elbow joint, for which his arm was amputated immediately above the wound. The stump appeared to do well for a time, but two months after the stump was attacked with pain, and continued at intervals until pus formed and an opening made for its exit. The pus was found to contain small particles of bone, and the pain was lessened for a time, but again returned, and continued at intervals until the patient was unable to endure it longer, when a second amputation was performed, in September, 1866, being two years and three months from the time of the first operation. The result was again ineffectual, as the pain and disease returned after a lapse of three months, which he endured until July, 1869, (the ligatures having been left in over two months may possibly have been the exciting cause,) when, from loss of sleep, occasioned by severe pain, he was unable to attend to his business. I was sent for, and found him considerably reduced, both in mind and body, nervous, slightly feverish, with quick pulse and very restless, complaining of pain in the bone extending to the shoulder joint. There was but little undue redness or swelling, and no external openings or appearances of any having existed. After a careful examination, I decided that nothing but the removal of the remaining portion of the humerus would prove effectual. Hence, on the following morning, I proceeded as follows: making an incision from the tip of the acromion process to the lower end of the diseased bone, which gave vent to a quantity of green and bloody pus, accompanied with a peculiar odor that always attends the escape of long pent up pus with diseased bone; it was with considerable difficulty that the bone was detached, there being anchylosis between the head of the bone and joint. The humerus being entirely removed, I was a little surprised to find the disease extended to the glenoid cavity and anterior border of scapula. An incision was now made from top of the acromion process along the anterior border to the anterior and inferior angle of the scapula, nearly half that bone being removed by the bone forceps, including the glenoid cavity; the diseased bone being all removed, the wound was well cleansed with a solution of carbolic acid, tents soaked in the same solution left in the cavity, the parts were bronght together with the ordinary suture, and cold water dressings sparingly applied. The patient done well, and returned to his home, in Iowa, eight days after the operation. A solution of carbolic acid was injected in the wound every day for three weeks after, to favor healthy granulation. The patient is yet perfectly free from any symptoms of the former difficulty, and healthier than ever before. Carbolic acid is, in my opinion, one of the most valuable remedies we possess for diseased bone, having had numerous cases to test the value of it.

